# Towards a Better Vision of Retinoic Acid Signaling during Eye Development

**DOI:** 10.3390/cells11030322

**Published:** 2022-01-19

**Authors:** Gregg Duester

**Affiliations:** Development, Aging, and Regeneration Program, Sanford Burnham Prebys Medical Discovery Institute, 10901 N. Torrey Pines Road, La Jolla, CA 92037, USA; duester@sbpdiscovery.org

**Keywords:** retinoic acid signaling, eye development, optic cup, cornea, transcriptional control

## Abstract

Retinoic acid (RA) functions as an essential signal for development of the vertebrate eye by controlling the transcriptional regulatory activity of RA receptors (RARs). During eye development, the optic vesicles and later the retina generate RA as a metabolite of vitamin A (retinol). Retinol is first converted to retinaldehyde by retinol dehydrogenase 10 (RDH10) and then to RA by all three retinaldehyde dehydrogenases (ALDH1A1, ALDH1A2, and ALDH1A3). In early mouse embryos, RA diffuses to tissues throughout the optic placode, optic vesicle, and adjacent mesenchyme to stimulate folding of the optic vesicle to form the optic cup. RA later generated by the retina is needed for further morphogenesis of the optic cup and surrounding perioptic mesenchyme; loss of RA at this stage leads to microphthalmia and cornea plus eyelid defects. RA functions by binding to nuclear RARs at RA response elements (RAREs) that either activate or repress transcription of key genes. Binding of RA to RARs regulates recruitment of transcriptional coregulators such as nuclear receptor coactivator (NCOA) or nuclear receptor corepressor (NCOR), which in turn control binding of the generic coactivator p300 or the generic corepressor PRC2. No genes have been identified as direct targets of RA signaling during eye development, so future studies need to focus on identifying such genes and their RAREs. Studies designed to learn how RA normally controls eye development in vivo will provide basic knowledge valuable for determining how developmental eye defects occur and for improving strategies to treat eye defects.

## 1. Introduction

**Figure 1:**
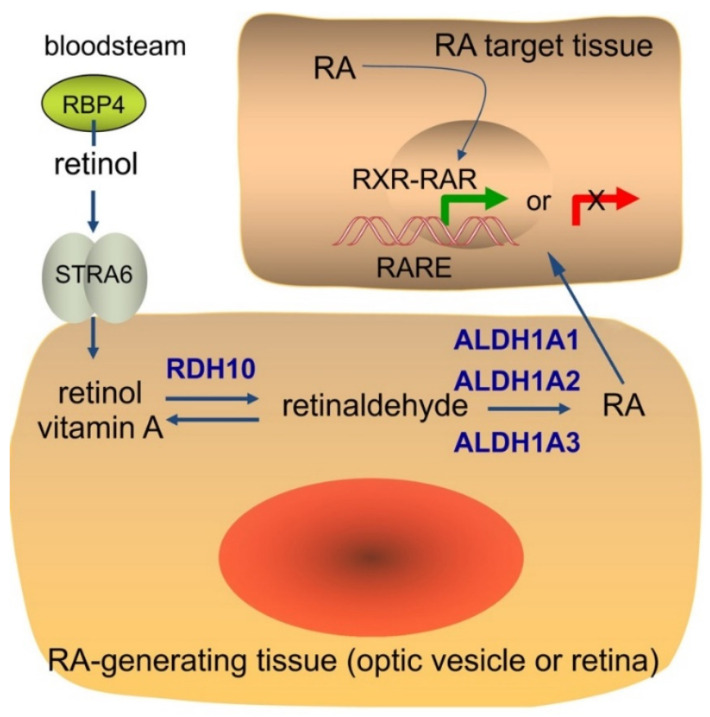
Generation of RA and control of transcriptional activation or repression of target genes.

Retinol, carried in the bloodstream by retinol-binding protein 4 (RBP4), is widely distributed in adult and embryonic tissues, and the membrane protein STRA6 facilitates cellular uptake of retinol carried by RBP4 [1]. However, the mechanism of RA signaling is dependent upon conversion of retinol to RA specifically in cells that possess RA-generating enzymes whose genes are expressed in a tissue-specific manner (Figure 1). During mouse eye development, retinol is oxidized to retinaldehyde by retinol dehydrogenase 10 (RDH10) expressed by *Rdh10* [2], and retinaldehyde is oxidized to RA by retinaldehyde dehydrogenases (RALDHs, i.e., ALDH1A1, ALDH1A2, and ALDH1A3) expressed by the *Aldh1a1*, *Aldh1a2*, and *Aldh1a3* genes [3,4,5]. RA released by RA-generating cells is taken up by surrounding target cells. RA controls transcription of key genes by regulating the activity of nuclear RA receptors (RARs), i.e., RARA, RARB, RARG encoded by *Rara*, *Rarb*, and *Rarg* in mouse. In order to regulate genes, RA binds to RARs that are bound to RA response elements (RAREs) as a heterodimer with retinoid X receptors (RXRs) [6,7]. Binding of RA to the RAR portion of the RAR-RXR heterodimer alters recruitment of nuclear receptor coactivators (NCOAs) known to activate transcription, or nuclear receptor corepressors (NCORs) known to repress transcription; also altered is recruitment of the p300 general coactivator or the PRC2 general corepressor [8,9,10]. Thus, RA regulates transcriptional activation or repression through RARE enhancers or RARE silencers [11].

RA controls critical functions during eye development in humans, mice, and zebrafish [12,13,14,15,16]. Human studies have associated mutations in four components of the RA signaling pathway (*RBP4*, *STRA6*, *ALDH1A3*, *RARB*) and two genes up-regulated by RA (*PITX2*, *FOXC1*) with anophthalmia/microphthalmia [12,13,14,15,16,17]. However, studies on mouse embryos will likely identify additional RA target genes. In mouse embryos, *Rdh10* is expressed at E8.5 in optic mesenchyme and at E9.5 onwards in the optic vesicle/cup [2]. *Aldh1a1* (*Raldh1*) is expressed in the dorsal retina from E9.5 onwards, *Aldh1a2* (*Raldh2*) is expressed in the optic mesenchyme from only E8.5 to E9.5, and *Aldh1a3* (*Raldh3*) is expressed in the ventral retina from E8.5 onwards [18]. RA is required for folding of the optic vesicle to form the optic cup and ventral retina as shown in *Rdh10*-/- embryos [2] and in *Aldh1a1/ldh1a2/Aldh1a3* triple knockouts [18]. *Aldh1a1/Aldh1a3* double knockouts form the optic cup (due to early *Aldh1a2* RA synthesis), but they exhibit excessive neural crest-derived perioptic mesenchyme growth and anterior segment defects (cornea and eyelid), and *Pitx2* and *Foxc1* were found to be down-regulated in perioptic mesenchyme [18,19]. *Pitx2* and *Foxc1* knockouts exhibit anterior segment eye defects, but as they still generate the optic cup [20,21], it is likely that RA regulates other genes very early needed for optic cup formation.

## 2. Requirement of Retinoic Acid for Optic Cup Formation

**Figure 2:**
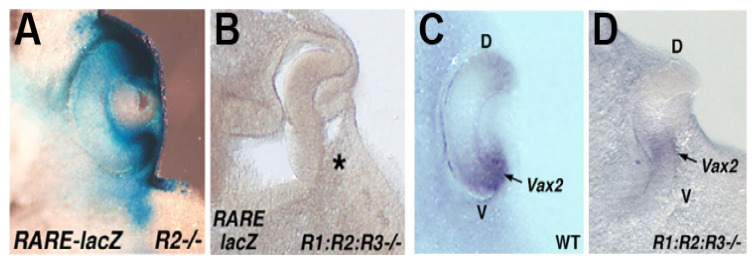
RA signaling is required during optic cup formation. (**A**) RA activity, detected by the *RARE-lacZ* transgene, and optic cup formation are both normal in E10.5 RA-rescued *Aldh1a2*-/- single knockout embryos (*R2*-/-); *Aldh1a2*-/- embryos do not develop beyond E8.5, but a single low-dose maternal treatment with exogenous RA at E7.5 results in growth to E10.5 and clearance of exogenous RA by E9.5 [25]; RA activity observed at E10.5 is due to expression of *Aldh1a1* and *Aldh1a3* in the optic vesicle [18]. (**B**) For E10.5 RA-rescued triple knockout embryos (*R1:R2:R3*-/-) one observes a lack of RA activity and a failure to form the optic cup; *, failure of ventral invagination of optic vesicle. (**C**,**D**) Another triple knockout (*R1:R2:R3*-/-) compared to wild type (WT) stained for *Vax2* mRNA (a marker of the ventral retina) also shows a failure in optic cup formation primarily due to a failure in ventral invagination of the optic vesicle. Shown are dorsoventral sections; adapted from [18].

During the earliest stage of eye development which begins at approximately E8.5 in mouse, the optic vesicles are formed as out-pocketings of the forebrain. Soon after that, the portion of the optic vesicle nearest to the head surface ectoderm invaginates back towards the forebrain to form an optic cup with a connection to the forebrain that will become the optic stalk. Early studies designed to reduce RA signaling, such as vitamin A deficiency in rat [22] and RAR double knockouts in mouse [23], showed that although the optic vesicle forms and undergoes optic cup formation which is complete at E10.5 in mouse, RA is required afterwards for further optic cup morphogenesis and proper formation of anterior eye structures adjacent to the optic cup such as cornea and eyelids. Although three RAR genes exist, which are all expressed in the early eye, triple RAR knockout studies have not been reported. However, RA function in mouse can also be examined genetically by knockout studies targeting RA-generating enzymes. At E9.5, when the optic vesicle begins to undergo optic cup formation, all three ALDH1A RA-generating enzymes are expressed, with *Aldh1a1* in the dorsal optic vesicle, *Aldh1a2* in the optic mesenchyme, and *Aldh1a3* in the ventral optic vesicle [18]. This triple redundancy, with *Aldh1a2*-/- embryos being stunted and not surviving beyond E8.5 [24], and with *Aldh1a3*-/- embryos not surviving past birth [4], thus hampers genetic studies to examine eye RA function. However, as *Aldh1a1*-/- mice survive as adults and are fertile [3], it was possible to generate adult mice that are *Aldh1a1*-/-/*Aldh1a2*+/-/*Aldh1a3*+/- and perform matings to generate E10.5 *Aldh1a1/Aldh1a2/Aldh1a3* triple knockout embryos (ratio of 1 in 16) to study optic cup formation in the absence of RA activity. In order to generate E10.5 embryos to study optic cup formation, this required treatment of the mother with one low dose of RA at E7.5 to prevent early lethality at E8.5 due to *Aldh1a2*-/- [25]; at E10.5 it was observed that all RA activity in the optic field was missing in the triple knockout and the optic cup did not form in the triple knockout while the eye was normal in control *Aldh1a2*-/- embryos due to the exogenous RA rescue performed at E7.5 plus the endogenous RA generated in the optic vesicles by *Aldh1a1* and *Aldh1a3* [18] (Figure 2). Thus, RA is required for invagination of the optic vesicle to form the optic cup; in particular, invagination of the ventral portion of the optic vesicle is inhibited by loss of RA signaling while some degree of dorsal invagination is still observed (Figure 2).

Subsequent to these mouse genetic loss-of-function studies, not much progress has been made on the mechanism through which RA controls optic cup formation. No direct RA target genes have been identified in the optic vesicle due to the difficulty of obtaining *Aldh1a1/Aldh1a2/Aldh1a3* triple knockouts [18]. Fortunately, RDH10 was found to be the only retinol dehydrogenase for the first step of RA synthesis in the eye (conversion of retinol to retinaldehyde); *Rdh10*-/- embryos survive to E10.5, have no RA activity detected in the optic field, and fail to undergo optic cup formation with loss of ventral invagination [2], similar to that observed in *Aldh1a1/Aldh1a2/Aldh1a3* triple knockouts. Further studies on *Rdh10*-/- embryos should allow progress to be made on the mechanism through which RA controls optic cup formation.

## 3. Requirement of Retinoic Acid for Morphogenesis of Anterior Eye Structures

Following optic cup formation at E10.5 in mouse, RA continues to be required for several stages of eye morphogenesis that result in maintenance of optic cup morphology and formation of anterior eye structures, particularly the cornea and eyelids. This later function of RA in eye morphogenesis was initially discovered by analysis of RAR double knockouts [23] and also by analysis of the RAR-alpha/RXR-alpha double knockout, thus revealing a requirement for RXR in eye RA signaling [26]. During these later stages, RA is generated only by *Aldh1a1* expressed in the dorsal retina and *Aldh1a3* expressed in the ventral retina as *Aldh1a2* expression is no longer observed after E9.5 [18]. Whereas *Aldh1a1* knockouts revealed no obvious eye defects due to compensation by *Aldh1a3*, and *Aldh1a3* knockouts exhibited only mild eye defects due to compensation by *Aldh1a1*, *Aldh1a1*/*Aldh1a3* double knockout embryos examined at E14.5 exhibited severe eye defects [18,19]. Interestingly, the *Aldh1a1*/*Aldh1a3* double knockout revealed that RA signaling is not required for formation of the retina or establishment or maintenance of dorsoventral patterning in the retina which had originally been proposed based on the dorsal and ventral expression patterns of *Aldh1a1* and *Aldh1a3* [18]. Instead, *Aldh1a1*/*Aldh1a3* double knockouts revealed that RA generated in the retina acts by diffusing outside of the optic cup to the nearby neural crest-derived perioptic mesenchyme to maintain optic cup shape and to control anterior eye formation [18,19]. RA was found to limit anterior invasion of perioptic mesenchyme during formation of corneal mesenchyme and eyelids which both show mesenchymal overgrowth in the *Aldh1a1*/*Aldh1a3* double knockout; such mesenchymal overgrowth also exerts mechanical force that results in abnormal optic cup morphology and position within the head [18,19]. These defects are consistent with the observation that loss of RA signaling reduced expression of *Pitx2* and *Foxc1* in perioptic mesenchyme [19]. These studies demonstrated that RA-generating enzymes function cell-nonautonomously to generate paracrine RA signals that guide eye morphogenetic movements in neighboring cells.

As the *Aldh1a3* knockout dies at birth, no studies were possible in the adult eye [4]. The initial studies on *Aldh1a1* knockout mice that survive to adulthood found no major eye defects in embryos and adults likely due to compensating expression of *Aldh1a3* in the ventral retina [3]. However, subsequent studies on the adult *Aldh1a1* knockout eye demonstrated a reduction in dorsal choroidal vascular development [27]; ventral choroidal development was normal in the *Aldh1a1* knockout presumably due to the action of *Aldh1a3* in the ventral retina.

**Figure 3:**
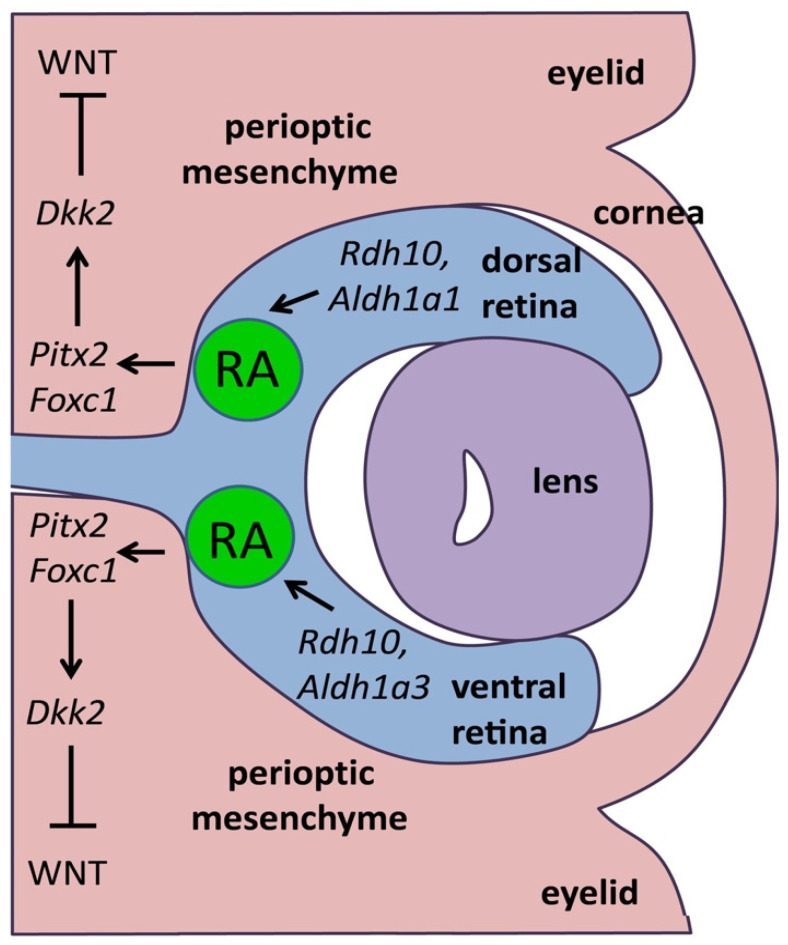
RA is generated in dorsal/ventral retina and diffuses to perioptic mesenchyme where it is required during anterior eye formation (cornea/eyelid) to activate *Pitx2* which then activates *Dkk2* that functions to repress WNT signaling to limit perioptic mesenchyme growth; adapted from [31].

RA also controls zebrafish eye development by activating *Pitx2* in neural crest-derived perioptic mesenchyme [28]. Additional mouse knockout studies confirmed that *Pitx2*-/- eyes exhibit excessive perioptic mesenchyme growth, plus *Pitx2* up-regulates *Dkk2* (encoding a WNT antagonist) in perioptic mesenchyme and *Dkk2*-/- eyes have a similar eye defect [20,29]. Further *Aldh1a1*/*Aldh1a3* double knockout studies showed that loss of RA generated in the retina results in loss of both *Pitx2* and *Dkk2* expression in perioptic mesenchyme plus increased expression of *Wnt5a*, thus demonstrating that RA activates *Pitx2* which then activates *Dkk2* to suppress WNT signaling and thus limit perioptic mesenchyme growth [30] (Figure 3).

*Pitx2* was found to possess a nearby RARE that is able to recruit RARs in eye ChIP studies, suggesting it may be a direct RA target gene important for anterior eye morphogenesis [30]. However, there has been no success in identifying any RA target gene for optic cup formation, and it remains unclear if *Pitx2* and *Foxc1* are direct RA target genes for later eye development or whether other late targets exist. Identification of direct RA target genes is difficult as thousands of RAREs are observed in the mouse and human genomes [32,33] plus the expression of thousands of genes is altered when RA is lost or added for any tissue or cell line examined [34,35]. In order to fully understand eye RA signaling, it will be essential to identify direct RA target genes that are activated or repressed in specific regions of the developing eye.

## 4. Identification of RA Direct Target Genes and Essential RAREs during Eye Formation

Identification of direct transcriptional targets of RA is difficult as loss or gain of RA signaling activity alters the expression of thousands of genes in cell lines or animals [34,35], with most genes probably being indirect targets of RA or regulated post-transcriptionally. As RA transcriptional control is associated with RAREs, identification of RAREs has been employed to identify direct RA target genes. A common approach is to find DNA sequences near RA-activated genes matching the RARE consensus that can activate transcription in reporter transgenes either in cell lines or transgenic animals. However, RAR chromatin immunoprecipitation (ChIP) analysis of mouse embryoid bodies found ~14,000 potential RAREs in the mouse genome [32,33]. Thus, it remains unclear which of these RAREs are needed to regulate genes during development since just a few of these RAREs have been shown to have specific enhancer activity in transgenic animals [7], and only three RAREs have been shown to result in developmental defects when deleted in mouse, i.e., the RARE enhancer activating *Hoxa1* in the hindbrain [36], a RARE enhancer activating *Cdx1* in the spinal cord [37], and a RARE silencer that represses caudal *Fgf8* in the developing trunk [38]. In one case, a RARE reported within intron 2 of *Tbx5* was reported to play a role in activation of *Tbx5* in the forelimb based on enhancer reporter transgene activity and RAR binding studies [39]; however, this RARE was shown to be unnecessary for *Tbx5* expression and forelimb budding when subjected to CRISPR deletion analysis by our laboratory [40]. A potentially redundant *Tbx5* forelimb enhancer was identified by others using an enhancer reporter transgene [41], but CRISPR studies showed that this enhancer is also unnecessary, plus a double knockout also had no affect showing the two enhancers are not redundant [40]. Thus, knockouts of RAREs and other types of DNA control elements sometimes result in validation of a required function; however, in many cases, presumed enhancers are nonessential. The disconnect is due to the fact that enhancer reporter transgenes are generated by joining a potential enhancer to a basal promoter upstream of a marker gene, then randomly inserting this into the genome; in this case, the enhancer is removed from its endogenous location and inserted into a foreign location close to a basal promoter, instead of within its normal location containing the gene and promoter it is proposed to control. It remains to be determined whether nonessential enhancers identified in transgene studies are redundant with yet more enhancers or whether they are pseudoenhancers not able to regulate expression of nearby genes [42].

These recent studies show that methods other than traditional transgenes are required to predict enhancers. Additionally, as transgenes are not generally useful to predict silencers, other methods are required to identify silencers. Genomic and epigenetic methods would be superior to the one gene at a time approach in order to allow global identification of important control elements. Advances in epigenetics have revealed chromatin modifications (such as H3K27ac associated with gene activation) and H3K27me3 (associated with gene repression) near transcription factor binding sites required for either gene activation (enhancers) or gene repression (silencers) [43,44,45]. The distances between genes and enhancers/silencers can be large, but these interactions occur within constrained chromatin domains known as topologically associated domains (TADs) that contain an average of 880 kb of DNA for ~2200 TADs [46]. TAD locations are conserved across cell types and mammalian species [46] and they are required for normal gene-enhancer interactions [47,48]. Therefore, RARE enhancers/silencers identified during optic cup formation and later eye morphogenesis will most likely control genes within the same TAD. Such RARE enhancers/silencers may be associated with H3K27ac or H3K27me3 marks controlled by RA signaling. In fact, recent studies have shown that identification of RA direct target genes can be accomplished by identifying genes with significant decreases or increases in expression when RA is missing that also have nearby RA-regulated deposition of H3K27ac (gene activation mark) or H3K27me3 (gene repression mark) associated with RAREs [11]. Such studies performed on trunk tissue from mouse E8.5 wild-type and *Aldh1a2*-/- embryos lacking RA synthesis resulted in the ability to take an RNA-seq list of 4298 genes shown to have significantly altered expression when RA is lost and reduce this down to 93 genes that also have RA-regulated deposition of nearby H3K27ac or H3K27me3 marks discovered using ChIP-seq, thus suggesting RA regulates their transcription. DNA sequence analysis of such RA-regulated H3K27ac/H3K27me3 ChIP-seq peaks revealed that 45 contain RAREs, providing evidence for the nearby genes being directly regulated by RA at the transcriptional level. This approach was validated by finding RAREs already known to be needed for development by previous knockout studies, i.e., deletions of *Hoxa1*, *Cdx1*, and *Fgf8* RAREs as described above. Many new candidates for direct RA target genes were found and knockouts of some (*Nr2f1, Nr2f2, Meis1, Meis2*) showed they are required for body axis or limb formation [11]. With this proof-of principle, similar studies on wild-type vs. RA-deficient eyes can be used to identify direct target genes for RA during eye formation. Such studies will provide vital information on the mechanisms utilized by RA to control transcription in the eye and will identify gene regulatory networks during eye formation. This knowledge will help determine how eye defects occur, identify new genes or enhancers/silencers that may be mutational targets causing human eye defects, and improve strategies to treat eye defects.
